# 1-(5-Hy­droxy-2,2,8,8-tetra­methyl-2*H*,8*H*-pyrano[2,3-*f*]chromen-6-yl)-3-(4-meth­oxy­phen­yl)prop-2-en-1-one

**DOI:** 10.1107/S1600536813005734

**Published:** 2013-03-02

**Authors:** Sunayna Pawar, Kaalin Gopaul, Thrineshan Moodley, Bernard Omondi, Neil Koorbanally

**Affiliations:** a School of Chemistry and Physics, University of KwaZulu-Natal, Durban 4000, South Africa

## Abstract

In the biologically active title compound, C_26_H_26_O_5_, the pyran ring of the chromene unit adopts a half-chair conformation. The C=C double bond of the propenone unit exhibits a *trans* conformation and the carbonyl group is *syn* conformation to the double bond. The dihedral angle between the benzene ring and the benzopyran­one moiety is 31.54 (4)°. The mol­ecular structure is stabilized by an intra­molecular C=O⋯H—O hydrogen bond.

## Related literature

For related structures, see: Bhattacharyya *et al.* (1999 ▶); Lee & Li (2007 ▶); Lin *et al.* (1992 ▶); Narender *et al.* (2005 ▶); Liu *et al.* (2005 ▶). For the biological activity of similar mol­ecules, see: Nicolaou *et al.* (2000 ▶); Dhar (1981 ▶). For bond lengths and angles in related structures, see: Bhattacharyya *et al.* (1999 ▶); Pawar *et al.* (2012 ▶).
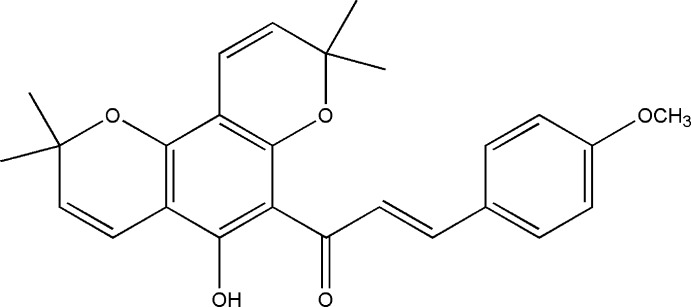


## Experimental

### 

#### Crystal data

C_26_H_26_O_5_
*M* *_r_* = 418.47
Monoclinic, 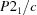

*a* = 9.6422 (2) Å
*b* = 12.0871 (3) Å
*c* = 18.4517 (4) Å
β = 99.228 (1)°
*V* = 2122.64 (8) Å^3^
*Z* = 4
Mo *K*α radiation
μ = 0.09 mm^−1^
*T* = 446 K
0.37 × 0.33 × 0.21 mm

#### Data collection

Bruker SMART APEXII CCD diffractometer
Absorption correction: multi-scan (*SADABS*; Bruker, 2008 ▶) *T*_min_ = 0.968, *T*_max_ = 0.981
68753 measured reflections
5126 independent reflections
4677 reflections with *I* > 2σ(*I*)
*R*_int_ = 0.020

#### Refinement

*R*[*F* ^2^ > 2σ(*F* ^2^)] = 0.037
*wR*(*F* ^2^) = 0.104
*S* = 1.01
5126 reflections
286 parameters
H-atom parameters constrained
Δρ_max_ = 0.38 e Å^−3^
Δρ_min_ = −0.23 e Å^−3^

### 

Data collection: *APEX2* (Bruker, 2008 ▶); cell refinement: *SAINT-Plus* (Bruker, 2008 ▶); data reduction: *SAINT-Plus* and *XPREP* (Bruker, 2008 ▶); program(s) used to solve structure: *SHELXS97* (Sheldrick, 2008 ▶); program(s) used to refine structure: *SHELXL97* (Sheldrick, 2008 ▶); molecular graphics: *ORTEP-3 for Windows* (Farrugia, 2012 ▶); software used to prepare material for publication: *WinGX* (Farrugia, 2012 ▶).

## Supplementary Material

Crystal structure: contains datablock(s) global, I. DOI: 10.1107/S1600536813005734/ff2099sup1.cif

Structure factors: contains datablock(s) I. DOI: 10.1107/S1600536813005734/ff2099Isup2.hkl

Supplementary material file. DOI: 10.1107/S1600536813005734/ff2099Isup3.cml

Additional supplementary materials:  crystallographic information; 3D view; checkCIF report

## Figures and Tables

**Table 1:** Hydrogen-bond geometry (Å, °)

*D*—H⋯*A*	*D*—H	H⋯*A*	*D*⋯*A*	*D*—H⋯*A*
O3—H3*A*⋯O2	0.82	1.72	2.4523 (10)	148

**Fig. 1.**
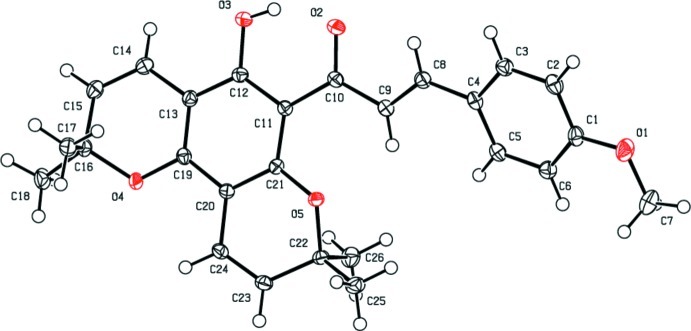
ORTEP diagram showing the molecular structure of the titled compound with atomic labelling scheme. Non-H atoms are drawn with 50% probability displacement ellipsoids and H atoms are shown as open circles.

## supplementary crystallographic information

### Comment

Chalcones are considered to be the primary precursors and constitute important intermediates in the synthesis of flavonoids. Pyranochromenechalcone is a core structure in various naturally active compounds (Nicolaou *et al.*, 2000). Chalcones are synthesized by the Claisen-Schmidt condensation of an aldehyde and ketone using a base as a catalyst, which is followed by dehydration. Chalcones possess antifungal, antioxidant,anti-inflammatory, antimalarial and antileishmanial activity amongst others (Dhar, 1981). With this in mind, a series of chalcones have been synthesized in our laboratory and their biological activity is currently under investigation.

The title compound is a 2-hydroxychalcone, with a central tricyclic core and a peripheral aromatic ring (Lee & Li 2007). The middle ring of the tricyclic core is fully substituted while the pyran rings have two unsubstituted sites. The pyran rings of the chromene unit forms a half chair conformation [Q = 0.3750 (10) Å, θ = 66.86 (15)° and ψ = 39.54 (17) °]. The C8— C9—C10—C11 torsion angle is 160.57 (9) Å, indicating a *trans* configuration of the double bond. This is supported by ^1^H NMR spectroscopy where a *trans* arrangement of the H atoms in the —CH = CH— group is evident by the coupling constant of *J* = 15.6 Hz. The carbonyl group is in a *cis* configuration with respect to the double bond (Liu *et al.* 2005). The dihedral angle between the phenyl ring and the benzopyranone moiety is 31.54 (4)°. Bond angles and lenghs are within normal ranges relative to reported compounds (Bhattacharyya *et al.*,1999; Pawar *et al.*, 2012).

### Experimental

Potassium hydroxide (112 mg, 0.7 mmol) and 4-methoxybenzaldehyde (95 mg, 0.7 mmol) was added to a solution of octandrenolone (150 mg, 0.5 mmol) in ethanol (10 ml) and water (2 ml) at room temperature. The reaction mixture was stirred for 48 h at room temperature. The solvent was then distilled off under reduced pressure and the residue dissolved in water (20 ml). The solution was then acidified with 2 N-HCl (20 ml) and the mixture extracted with ethyl acetate (3 × 30 ml), washed with water, and dried over anhydrous MgSO_4_. Removal of the solvent followed by flash column chromatography on silica gel gave the pure compound (178 mg, 85%) as a brown solid with a m.p. of 125 - 126 *^o^*C.

^1^HNMR (400 MHz, CDCl_3_): 14.49 (1*H*, s), 8.00 (1*H*, d, *J* = 15.56 Hz), 7.76 (1*H*, d, *J* = 15.56 Hz), 7.55 (2*H*, d, *J* = 8.32 Hz), 6.93 (2*H*, d, *J* = 8.32 Hz), 6.69 (1*H*, d, *J* = 10.0 Hz), 6.61 (1*H*,d, *J* = 10.0 Hz), 5.47 (2*H*, d, *J*= 10.0 Hz), 3.85 (3*H*, s), 1.55 (6*H*, s), 1.45 (6*H*, s)

^13^C NMR (100 MHz, CDCl_3_): 193.0, 161.7, 161.5, 156.3, 155.3, 142.4, 130.1, 128.6, 125.5, 125.4, 124.9, 116.8, 116.5, 114.5, 106.1, 102.8, 102.7, 78.3, 78.4, 55.6, 28.3, 28.6

### Refinement

Carbon-bound H-atoms were placed in calculated positions [C—H = 0.96 A for Me H atoms and 0.93 A for aromatic H atoms; *U*iso(H) = 1.2*U*eq(C) (1.5 for Me groups)] and were included in the refinement in the riding model approximation. The O—H H-atom was located in a difference map and also placed in a calculated position O—H = 0.82 A (*U*iso(H) = 1.2*U*eq(O).

### Crystal data

**Table:**

C_26_H_26_O_5_	*F*(000) = 888
*M**_r_* = 418.47	*D*_x_ = 1.309 Mg m^−^^3^
Monoclinic, *P*2_1_/*c*	Mo *K*α radiation, λ = 0.71073 Å
Hall symbol: -P 2ybc	Cell parameters from 71516 reflections
*a* = 9.6422 (2) Å	θ = 2.0–28°
*b* = 12.0871 (3) Å	µ = 0.09 mm^−^^1^
*c* = 18.4517 (4) Å	*T* = 446 K
β = 99.228 (1)°	Block, brown
*V* = 2122.64 (8) Å^3^	0.37 × 0.33 × 0.21 mm
*Z* = 4	

### Data collection

**Table:**

Bruker SMART APEXII CCD diffractometer	4677 reflections with *I* > 2σ(*I*)
Graphite monochromator	*R*_int_ = 0.020
φ and ω scans	θ_max_ = 28°, θ_min_ = 2.0°
Absorption correction: multi-scan (*SADABS*; Bruker, 2008)	*h* = −12→12
*T*_min_ = 0.968, *T*_max_ = 0.981	*k* = −15→15
68753 measured reflections	*l* = −24→24
5126 independent reflections	

### Refinement

**Table:**

Refinement on *F*^2^	Primary atom site location: structure-invariant direct methods
Least-squares matrix: full	Secondary atom site location: difference Fourier map
*R*[*F*^2^ > 2σ(*F*^2^)] = 0.037	Hydrogen site location: inferred from neighbouring sites
*wR*(*F*^2^) = 0.104	H-atom parameters constrained
*S* = 1.01	*w* = 1/[σ^2^(*F*_o_^2^) + (0.0564*P*)^2^ + 0.9316*P*] where *P* = (*F*_o_^2^ + 2*F*_c_^2^)/3
5126 reflections	(Δ/σ)_max_ = 0.003
286 parameters	Δρ_max_ = 0.38 e Å^−^^3^
0 restraints	Δρ_min_ = −0.23 e Å^−^^3^

### Special details

**Table:**

Experimental. Carbon-bound H-atoms were placed in calculated positions [C—H = 0.96 Å for Me H atoms and 0.93 Å for aromatic H atoms; *U*_iso_(H) = 1.2*U*_eq_(C) (1.5 for Me groups)] and were included in the refinement in the riding model approximation. The O—H H-atom was located in a difference map and also placed in calculated position O—H = 0.84 Å (*U*_iso_(H) = 1.2*U*_eq_(O).
Geometry. All e.s.d.'s (except the e.s.d. in the dihedral angle between two l.s. planes) are estimated using the full covariance matrix. The cell e.s.d.'s are taken into account individually in the estimation of e.s.d.'s in distances, angles and torsion angles; correlations between e.s.d.'s in cell parameters are only used when they are defined by crystal symmetry. An approximate (isotropic) treatment of cell e.s.d.'s is used for estimating e.s.d.'s involving l.s. planes.
Refinement. Refinement of *F*^2^ against ALL reflections. The weighted *R*-factor *wR* and goodness of fit *S* are based on *F*^2^, conventional *R*-factors *R* are based on *F*, with *F* set to zero for negative *F*^2^. The threshold expression of *F*^2^ > σ(*F*^2^) is used only for calculating *R*-factors(gt) *etc*. and is not relevant to the choice of reflections for refinement. *R*-factors based on *F*^2^ are statistically about twice as large as those based on *F*, and *R*- factors based on ALL data will be even larger. >>> The Following Model ALERT was generated - (Acta-Mode) <<< Format: alert-number_ALERT_alert-type_alert-level text 918_ALERT_3_C Reflection(*s*) # with *I*(obs) much smaller *I*(calc) 1 NOTED:

### Fractional atomic coordinates and isotropic or equivalent isotropic displacement parameters (Å^2^)

**Table:**

	*x*	*y*	*z*	*U*_iso_*/*U*_eq_	
C1	0.14616 (10)	0.12942 (8)	0.45580 (6)	0.0187 (2)	
C2	0.12410 (11)	0.11703 (8)	0.52813 (6)	0.0207 (2)	
H2	0.1144	0.0468	0.5473	0.025*	
C3	0.11675 (11)	0.20968 (9)	0.57126 (6)	0.0192 (2)	
H3	0.1007	0.201	0.6193	0.023*	
C4	0.13298 (10)	0.31683 (8)	0.54411 (5)	0.01655 (19)	
C5	0.15481 (10)	0.32699 (8)	0.47122 (6)	0.0179 (2)	
H5	0.1655	0.397	0.452	0.022*	
C6	0.16087 (11)	0.23495 (9)	0.42712 (6)	0.0190 (2)	
H6	0.1747	0.2434	0.3787	0.023*	
C7	0.17381 (13)	0.04268 (10)	0.34274 (6)	0.0282 (2)	
H7A	0.0998	0.0862	0.3156	0.042*	
H7B	0.1735	−0.0298	0.3215	0.042*	
H7C	0.2626	0.0777	0.341	0.042*	
C8	0.12434 (10)	0.41146 (8)	0.59236 (5)	0.01725 (19)	
H8	0.096	0.3965	0.6372	0.021*	
C9	0.15272 (10)	0.51777 (8)	0.57928 (5)	0.01690 (19)	
H9	0.1821	0.538	0.5355	0.02*	
C10	0.13665 (10)	0.60171 (8)	0.63516 (5)	0.01499 (18)	
C11	0.20284 (9)	0.71156 (8)	0.63883 (5)	0.01365 (18)	
C12	0.17283 (10)	0.78670 (8)	0.69429 (5)	0.01453 (18)	
C13	0.23344 (10)	0.89200 (8)	0.70296 (5)	0.01503 (19)	
C14	0.19923 (10)	0.97192 (8)	0.75649 (6)	0.0185 (2)	
H14	0.1228	0.9603	0.7806	0.022*	
C15	0.27897 (11)	1.06184 (9)	0.77042 (6)	0.0200 (2)	
H15	0.2537	1.1163	0.8014	0.024*	
C16	0.40971 (10)	1.07586 (8)	0.73628 (5)	0.01676 (19)	
C17	0.53457 (11)	1.01888 (9)	0.78265 (6)	0.0195 (2)	
H17A	0.5159	0.9411	0.7853	0.029*	
H17B	0.5492	1.0497	0.8312	0.029*	
H17C	0.6172	1.03	0.7606	0.029*	
C18	0.44156 (12)	1.19660 (9)	0.72296 (6)	0.0227 (2)	
H18A	0.5228	1.2014	0.6991	0.034*	
H18B	0.4595	1.235	0.7691	0.034*	
H18C	0.3625	1.2297	0.6923	0.034*	
C19	0.32725 (10)	0.92351 (8)	0.65633 (5)	0.01412 (18)	
C20	0.35864 (10)	0.85484 (8)	0.60002 (5)	0.01394 (18)	
C21	0.29703 (10)	0.74988 (8)	0.59257 (5)	0.01330 (18)	
C22	0.38220 (10)	0.72295 (8)	0.47666 (5)	0.01553 (19)	
C23	0.47626 (10)	0.82157 (8)	0.49591 (5)	0.01718 (19)	
H23	0.5448	0.8386	0.4675	0.021*	
C24	0.46159 (10)	0.88436 (8)	0.55330 (5)	0.01615 (19)	
H24	0.517	0.9471	0.5637	0.019*	
C25	0.45997 (12)	0.62799 (9)	0.44730 (6)	0.0212 (2)	
H25A	0.5382	0.6066	0.4836	0.032*	
H25B	0.4935	0.6511	0.4034	0.032*	
H25C	0.3975	0.5663	0.4364	0.032*	
C26	0.25010 (12)	0.75443 (9)	0.42370 (6)	0.0224 (2)	
H26A	0.1905	0.6908	0.414	0.034*	
H26B	0.2758	0.7808	0.3786	0.034*	
H26C	0.2008	0.8116	0.4452	0.034*	
O1	0.15230 (9)	0.03378 (6)	0.41723 (5)	0.02628 (18)	
O2	0.06220 (8)	0.57627 (6)	0.68277 (4)	0.02028 (16)	
O3	0.08410 (8)	0.75943 (6)	0.74064 (4)	0.01852 (16)	
H3A	0.0569	0.6957	0.7326	0.028*	
O4	0.38941 (7)	1.02473 (6)	0.66293 (4)	0.01655 (15)	
O5	0.33812 (7)	0.67778 (6)	0.54356 (4)	0.01567 (15)	

### Atomic displacement parameters (Å^2^)

**Table:**

	*U*^11^	*U*^22^	*U*^33^	*U*^12^	*U*^13^	*U*^23^
C1	0.0172 (4)	0.0153 (5)	0.0231 (5)	0.0006 (3)	0.0014 (4)	−0.0037 (4)
C2	0.0228 (5)	0.0137 (4)	0.0249 (5)	−0.0015 (4)	0.0021 (4)	0.0022 (4)
C3	0.0206 (5)	0.0182 (5)	0.0186 (5)	−0.0020 (4)	0.0025 (4)	0.0018 (4)
C4	0.0138 (4)	0.0148 (4)	0.0204 (5)	−0.0014 (3)	0.0009 (3)	−0.0007 (4)
C5	0.0176 (4)	0.0145 (4)	0.0213 (5)	−0.0017 (3)	0.0022 (4)	0.0014 (4)
C6	0.0192 (5)	0.0191 (5)	0.0186 (5)	−0.0006 (4)	0.0027 (4)	−0.0001 (4)
C7	0.0337 (6)	0.0257 (6)	0.0239 (5)	0.0054 (5)	0.0010 (4)	−0.0075 (4)
C8	0.0166 (4)	0.0175 (5)	0.0175 (4)	−0.0014 (3)	0.0022 (3)	−0.0001 (4)
C9	0.0166 (4)	0.0170 (5)	0.0173 (4)	−0.0031 (3)	0.0035 (3)	−0.0013 (4)
C10	0.0144 (4)	0.0145 (4)	0.0158 (4)	−0.0011 (3)	0.0018 (3)	0.0015 (3)
C11	0.0140 (4)	0.0131 (4)	0.0139 (4)	−0.0006 (3)	0.0022 (3)	0.0000 (3)
C12	0.0134 (4)	0.0162 (4)	0.0142 (4)	0.0003 (3)	0.0029 (3)	0.0007 (3)
C13	0.0150 (4)	0.0147 (4)	0.0155 (4)	0.0002 (3)	0.0025 (3)	−0.0017 (3)
C14	0.0177 (4)	0.0188 (5)	0.0200 (5)	0.0013 (4)	0.0062 (4)	−0.0030 (4)
C15	0.0224 (5)	0.0173 (5)	0.0213 (5)	0.0013 (4)	0.0064 (4)	−0.0056 (4)
C16	0.0215 (5)	0.0131 (4)	0.0157 (4)	−0.0023 (3)	0.0030 (3)	−0.0033 (3)
C17	0.0214 (5)	0.0187 (5)	0.0181 (5)	−0.0015 (4)	0.0021 (4)	−0.0009 (4)
C18	0.0303 (5)	0.0136 (5)	0.0239 (5)	−0.0037 (4)	0.0029 (4)	−0.0018 (4)
C19	0.0154 (4)	0.0117 (4)	0.0147 (4)	−0.0003 (3)	0.0006 (3)	0.0006 (3)
C20	0.0152 (4)	0.0136 (4)	0.0131 (4)	−0.0008 (3)	0.0024 (3)	0.0008 (3)
C21	0.0144 (4)	0.0131 (4)	0.0123 (4)	0.0008 (3)	0.0018 (3)	−0.0002 (3)
C22	0.0191 (4)	0.0150 (4)	0.0135 (4)	−0.0009 (3)	0.0057 (3)	0.0002 (3)
C23	0.0183 (4)	0.0166 (4)	0.0177 (4)	−0.0022 (3)	0.0062 (3)	0.0018 (4)
C24	0.0175 (4)	0.0144 (4)	0.0168 (4)	−0.0031 (3)	0.0035 (3)	0.0017 (3)
C25	0.0270 (5)	0.0171 (5)	0.0220 (5)	0.0009 (4)	0.0112 (4)	−0.0011 (4)
C26	0.0233 (5)	0.0236 (5)	0.0193 (5)	0.0004 (4)	0.0000 (4)	0.0003 (4)
O1	0.0366 (4)	0.0160 (4)	0.0266 (4)	0.0009 (3)	0.0062 (3)	−0.0054 (3)
O2	0.0237 (4)	0.0179 (4)	0.0213 (4)	−0.0052 (3)	0.0097 (3)	−0.0003 (3)
O3	0.0199 (3)	0.0182 (3)	0.0192 (3)	−0.0042 (3)	0.0087 (3)	−0.0021 (3)
O4	0.0223 (3)	0.0123 (3)	0.0153 (3)	−0.0039 (3)	0.0036 (3)	−0.0020 (2)
O5	0.0206 (3)	0.0128 (3)	0.0152 (3)	−0.0019 (2)	0.0076 (3)	−0.0010 (2)

### Geometric parameters (Å, º)

**Table:**

C1—O1	1.3638 (12)	C15—C16	1.5064 (14)
C1—C2	1.3927 (15)	C15—H15	0.93
C1—C6	1.3969 (14)	C16—O4	1.4722 (11)
C2—C3	1.3823 (15)	C16—C18	1.5194 (14)
C2—H2	0.93	C16—C17	1.5249 (14)
C3—C4	1.4063 (14)	C17—H17A	0.96
C3—H3	0.93	C17—H17B	0.96
C4—C5	1.3997 (14)	C17—H17C	0.96
C4—C8	1.4601 (14)	C18—H18A	0.96
C5—C6	1.3851 (14)	C18—H18B	0.96
C5—H5	0.93	C18—H18C	0.96
C6—H6	0.93	C19—O4	1.3593 (11)
C7—O1	1.4266 (14)	C19—C20	1.4004 (13)
C7—H7A	0.96	C20—C21	1.3983 (13)
C7—H7B	0.96	C20—C24	1.4595 (13)
C7—H7C	0.96	C21—O5	1.3599 (11)
C8—C9	1.3438 (14)	C22—O5	1.4733 (11)
C8—H8	0.93	C22—C23	1.5054 (13)
C9—C10	1.4725 (13)	C22—C25	1.5177 (14)
C9—H9	0.93	C22—C26	1.5241 (14)
C10—O2	1.2585 (12)	C23—C24	1.3288 (14)
C10—C11	1.4699 (13)	C23—H23	0.93
C11—C21	1.4201 (13)	C24—H24	0.93
C11—C12	1.4321 (13)	C25—H25A	0.96
C12—O3	1.3441 (11)	C25—H25B	0.96
C12—C13	1.3990 (13)	C25—H25C	0.96
C13—C19	1.3975 (13)	C26—H26A	0.96
C13—C14	1.4568 (13)	C26—H26B	0.96
C14—C15	1.3323 (14)	C26—H26C	0.96
C14—H14	0.93	O3—H3A	0.82
			
O1—C1—C2	115.78 (9)	C15—C16—C17	110.67 (8)
O1—C1—C6	124.11 (9)	C18—C16—C17	111.27 (8)
C2—C1—C6	120.11 (9)	C16—C17—H17A	109.5
C3—C2—C1	119.63 (9)	C16—C17—H17B	109.5
C3—C2—H2	120.2	H17A—C17—H17B	109.5
C1—C2—H2	120.2	C16—C17—H17C	109.5
C2—C3—C4	121.47 (9)	H17A—C17—H17C	109.5
C2—C3—H3	119.3	H17B—C17—H17C	109.5
C4—C3—H3	119.3	C16—C18—H18A	109.5
C5—C4—C3	117.75 (9)	C16—C18—H18B	109.5
C5—C4—C8	123.32 (9)	H18A—C18—H18B	109.5
C3—C4—C8	118.93 (9)	C16—C18—H18C	109.5
C6—C5—C4	121.40 (9)	H18A—C18—H18C	109.5
C6—C5—H5	119.3	H18B—C18—H18C	109.5
C4—C5—H5	119.3	O4—C19—C13	120.65 (8)
C5—C6—C1	119.64 (9)	O4—C19—C20	117.08 (8)
C5—C6—H6	120.2	C13—C19—C20	122.25 (9)
C1—C6—H6	120.2	C21—C20—C19	118.14 (8)
O1—C7—H7A	109.5	C21—C20—C24	118.84 (8)
O1—C7—H7B	109.5	C19—C20—C24	122.80 (9)
H7A—C7—H7B	109.5	O5—C21—C20	118.84 (8)
O1—C7—H7C	109.5	O5—C21—C11	118.25 (8)
H7A—C7—H7C	109.5	C20—C21—C11	122.65 (8)
H7B—C7—H7C	109.5	O5—C22—C23	109.86 (7)
C9—C8—C4	127.33 (9)	O5—C22—C25	104.14 (7)
C9—C8—H8	116.3	C23—C22—C25	111.69 (8)
C4—C8—H8	116.3	O5—C22—C26	107.81 (8)
C8—C9—C10	119.06 (9)	C23—C22—C26	111.31 (8)
C8—C9—H9	120.5	C25—C22—C26	111.71 (8)
C10—C9—H9	120.5	C24—C23—C22	120.21 (9)
O2—C10—C11	118.73 (9)	C24—C23—H23	119.9
O2—C10—C9	117.15 (9)	C22—C23—H23	119.9
C11—C10—C9	124.12 (8)	C23—C24—C20	120.03 (9)
C21—C11—C12	116.40 (8)	C23—C24—H24	120
C21—C11—C10	125.57 (8)	C20—C24—H24	120
C12—C11—C10	118.02 (8)	C22—C25—H25A	109.5
O3—C12—C13	116.57 (8)	C22—C25—H25B	109.5
O3—C12—C11	121.42 (9)	H25A—C25—H25B	109.5
C13—C12—C11	122.01 (9)	C22—C25—H25C	109.5
C19—C13—C12	118.51 (9)	H25A—C25—H25C	109.5
C19—C13—C14	118.43 (9)	H25B—C25—H25C	109.5
C12—C13—C14	123.02 (9)	C22—C26—H26A	109.5
C15—C14—C13	119.03 (9)	C22—C26—H26B	109.5
C15—C14—H14	120.5	H26A—C26—H26B	109.5
C13—C14—H14	120.5	C22—C26—H26C	109.5
C14—C15—C16	120.46 (9)	H26A—C26—H26C	109.5
C14—C15—H15	119.8	H26B—C26—H26C	109.5
C16—C15—H15	119.8	C1—O1—C7	117.64 (9)
O4—C16—C15	109.90 (8)	C12—O3—H3A	109.5
O4—C16—C18	104.77 (8)	C19—O4—C16	116.87 (7)
C15—C16—C18	112.34 (8)	C21—O5—C22	118.27 (7)
O4—C16—C17	107.63 (8)		
			
O1—C1—C2—C3	179.87 (9)	C14—C13—C19—O4	2.68 (14)
C6—C1—C2—C3	0.06 (15)	C12—C13—C19—C20	2.16 (14)
C1—C2—C3—C4	−0.88 (15)	C14—C13—C19—C20	−175.54 (9)
C2—C3—C4—C5	0.96 (15)	O4—C19—C20—C21	179.39 (8)
C2—C3—C4—C8	−179.91 (9)	C13—C19—C20—C21	−2.33 (14)
C3—C4—C5—C6	−0.24 (14)	O4—C19—C20—C24	4.86 (14)
C8—C4—C5—C6	−179.33 (9)	C13—C19—C20—C24	−176.85 (9)
C4—C5—C6—C1	−0.55 (15)	C19—C20—C21—O5	−172.89 (8)
O1—C1—C6—C5	−179.15 (9)	C24—C20—C21—O5	1.87 (13)
C2—C1—C6—C5	0.65 (15)	C19—C20—C21—C11	1.10 (14)
C5—C4—C8—C9	−9.13 (16)	C24—C20—C21—C11	175.85 (9)
C3—C4—C8—C9	171.79 (10)	C12—C11—C21—O5	174.23 (8)
C4—C8—C9—C10	179.61 (9)	C10—C11—C21—O5	−4.63 (14)
C8—C9—C10—O2	−18.82 (14)	C12—C11—C21—C20	0.21 (14)
C8—C9—C10—C11	160.57 (9)	C10—C11—C21—C20	−178.65 (9)
O2—C10—C11—C21	175.47 (9)	O5—C22—C23—C24	−29.29 (12)
C9—C10—C11—C21	−3.91 (15)	C25—C22—C23—C24	−144.33 (10)
O2—C10—C11—C12	−3.37 (13)	C26—C22—C23—C24	90.05 (11)
C9—C10—C11—C12	177.24 (9)	C22—C23—C24—C20	2.94 (15)
C21—C11—C12—O3	179.36 (8)	C21—C20—C24—C23	12.64 (14)
C10—C11—C12—O3	−1.69 (14)	C19—C20—C24—C23	−172.87 (9)
C21—C11—C12—C13	−0.39 (14)	C2—C1—O1—C7	179.59 (9)
C10—C11—C12—C13	178.56 (8)	C6—C1—O1—C7	−0.61 (15)
O3—C12—C13—C19	179.49 (8)	C13—C19—O4—C16	27.90 (12)
C11—C12—C13—C19	−0.75 (14)	C20—C19—O4—C16	−153.79 (8)
O3—C12—C13—C14	−2.92 (14)	C15—C16—O4—C19	−43.92 (11)
C11—C12—C13—C14	176.84 (9)	C18—C16—O4—C19	−164.82 (8)
C19—C13—C14—C15	−14.31 (14)	C17—C16—O4—C19	76.67 (10)
C12—C13—C14—C15	168.10 (10)	C20—C21—O5—C22	−31.54 (12)
C13—C14—C15—C16	−5.24 (15)	C11—C21—O5—C22	154.21 (8)
C14—C15—C16—O4	33.00 (13)	C23—C22—O5—C21	43.94 (11)
C14—C15—C16—C18	149.22 (10)	C25—C22—O5—C21	163.69 (8)
C14—C15—C16—C17	−85.74 (12)	C26—C22—O5—C21	−77.52 (10)
C12—C13—C19—O4	−179.62 (8)		

### Hydrogen-bond geometry (Å, º)

**Table:**

*D*—H···*A*	*D*—H	H···*A*	*D*···*A*	*D*—H···*A*
O3—H3*A*···O2	0.82	1.72	2.4523 (10)	148
